# Paediatric Burns of the Hand: Our Experience Over Three Years

**DOI:** 10.7759/cureus.18970

**Published:** 2021-10-22

**Authors:** Dujanah S Bhatti, Rafsan Chowdhury, Kok Kiong Ang, Jennifer Greenhowe

**Affiliations:** 1 Plastic and Reconstructive Surgery, Aberdeen Royal Infirmary, Aberdeen, GBR; 2 Plastic Surgery, University of Aberdeen, Aberdeen, GBR; 3 Plastic Surgery, NHS Grampian, Aberdeen, GBR; 4 Plastic and Reconstructive Surgery, Royal Aberdeen Children's Hospital, Aberdeen, GBR

**Keywords:** hand injuries, paediatic, burn cases, pediatric plastic surgery, paediatric hand, burns wound

## Abstract

Background and aim

Contact burn injuries to the hand are common in the paediatric population, with the most common aetiology involving touching hot surfaces in the household. The hand is also often involved in paediatric scald injuries. The aim of this study was to determine the different presentations of hand burn injuries and analyse the outcomes in the paediatric population at Royal Aberdeen Children’s Hospital (RACH).

Methods

Anonymised clinic data for paediatric patients with hand burns presenting to our burn centre from 2017 to 2020 were retrospectively reviewed. A total of 52 patients (65 affected hands) were included in the study. Clinic letters stored on NHS Grampian’s electronic patient record system were reviewed for burn surface area, time to healing, management measures including medications prescribed and sequelae of the burn injury.

Results

The average patient age was three years and four months old. There were 31 male patients and 21 female patients. Paediatric hand burns were most commonly confined to the palm only, followed by the fingers only. Contact with a hob was the most common aetiology, followed by scald burns. The average time to healing was 10 days (range 2-28 days). No correlation was found between length of stay on initial hospital admission and time to complete healing. A total of 86.5% (n=45) of patients were managed with dressings and 13.5% (n=7) of patients underwent surgical management. Of these seven patients, four had surgical debridement of burn tissue, washout, and dressing, and the remaining three had an excision and grafting with thick split-thickness skin grafts. Of these three patients, one patient had to undergo secondary reconstruction with a full-thickness skin graft.

Conclusion

It has been found that most patients in this study completely healed with primarily conservative measures of dressing care and regular check-ups. Isolated hand burns in the paediatric population present a low rate of sequelae and palms are the most common area of burn injury in this demographic.

## Introduction

Children are curious by nature and as soon they become independently mobile, they start to use their hands to explore their environment. This process is a crucial step in their overall development. Inevitably, they will come into contact with items within their surroundings that can result in injuries.

Children are one of the most vulnerable groups to sustain burn injuries. In the paediatric population, the fifth most common cause of non-fatal injuries is burns [1]. Only 4% of total body surface area accounts for the skin of hands. Injury to the hands/digits occurs in 35% of the burn cases in patients ages <5 and 24% in patients ages 5-16 years due to their involvement in most of the everyday activities with contact hand thermal injuries being common in children especially those under the age of 16 years [2]. Studies have highlighted that most of these burns occur within the household, generally related to touching hot surfaces [3-5]. The management of thermal injuries in children is largely similar to adults. However, children lack the thick glabrous skin of adults, therefore, thermal hand injuries can be deep and result in not only significant physical consequences but also long-lasting psychological effects [6]. Indeed, an extensive retrospective cohort study in Western Australia found that post-burn mental health admissions are much higher compared to a similarly aged group of uninjured children [2].

Thermal injuries involving hands can be complicated and require proper management plans to protect function and appearance. For a child, thermal injuries can be overwhelming, and management of these injuries can result in pain and distress. Some of these children will face permanent scarring and subsequently reduced function as well.

Given that burn injuries to the hand comprise a significant number of paediatric burn injury hospital admissions, we hope to share our unit’s experience regarding such injuries to aid the progress towards the development of outcome predictors, optimal management pathways for both the injury and possible psychological trauma and prevention methods.

## Materials and methods

Anonymised clinical data for paediatric patients with hand burns to present to our burn unit at Royal Aberdeen Children's Hospital (RACH) from 2017 to 2020 were collected in our retrospective cohort study of assessing pediatric burn injuries to the hand over three years. Our inclusion criteria included children younger than 16 years of age who presented to the RACH emergency department or were referred to the Plastic Surgery Unit with burn injures to the hands. Children suffering from burn injuries other than the hands or suffering from any systemic illness and hand burns not referred to the plastic surgery team were excluded from the study. Data were collected from patients' records retrieved from the Department of Plastic Surgery based on our criteria from the day of assessment in ER or clinic till the last follow-up appointment.

Basic demographics including date of birth, gender, and age at the time of injury were recorded. A total of 52 patients (65 affected hands) were included. The clinic letters stored on NHS Grampian’s electronic patient record system were analysed for the mechanism of injury, burn surface area, follow-up time to healing, management measures including medications, and sequelae. Statistical analysis was performed using the Statistical Package for the Social Sciences (SPSS) software v. 26 (IBM Corporation, Armonk, NY) and with the help of Microsoft Excel (2007).

## Results

Fifty-two patients sustained isolated burns over hands during the study period. The mean patient age at the time of injury was three years and four months old (two months to nine years). There were 31 male patients and 21 female patients. Patients presented to the burn unit at an average of 7.8 days following injury, after being assessed and managed in the acute emergency unit following the injury. Mean follow-up was 1.8 weeks (one week to four weeks). Length of hospital admission varied depending on burn sustained, ranging from discharge on the same day, with daily dressing, to nine days post-surgery.

**Figure 1:**
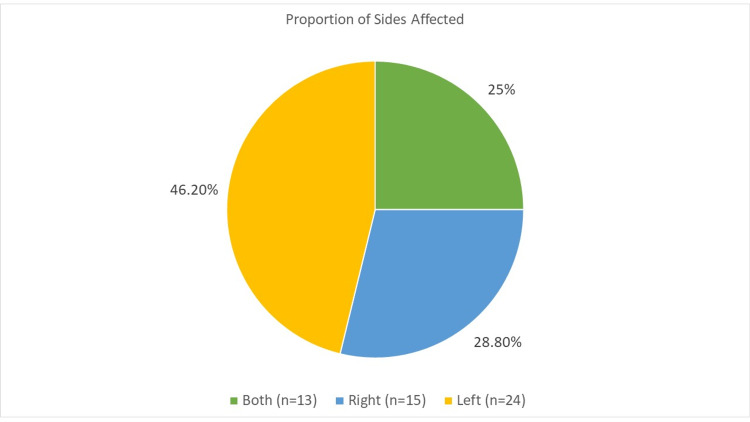
Percentage of hand affected (right, left, or both) in burn injury.

**Figure 2:**
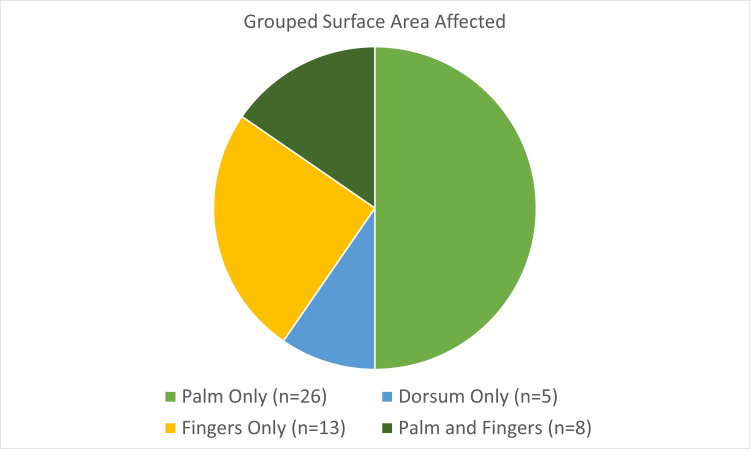
Area of hand affected by burn injury. Pie chart comparing palm only, dorsum of hand only, fingers only, and both palm and fingers involvement. Pie chart comparing palm only, dorsum of hand only, fingers only, and both palm and fingers involvement.

Both hands sustained burns in 25% of cases, while in 46.2% only the left hand was affected and in 28.8% it was the right hand (Figure 1). In 50% of cases only the palm was involved, in 25% only fingers, in 10% the dorsum, and 15% involved the palm and fingers (Figure 2).

**Figure 3:**
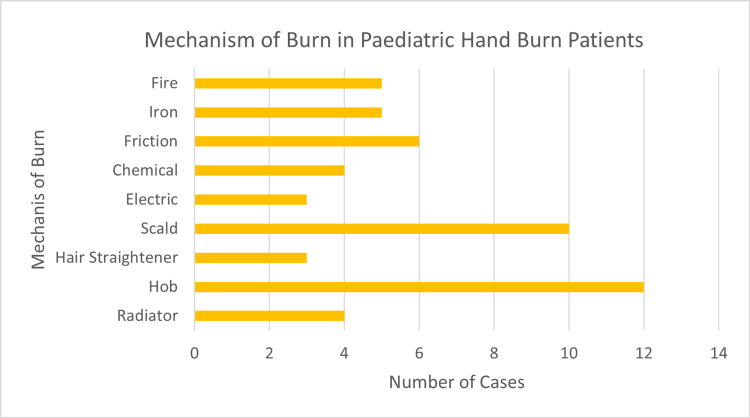
Horizontal bar graph comparing the mechanism of injury in our study cohort.

The most common mechanisms of injury were found to be on the hob (n=12) and scald injuries (n=10; Figure 3). Other mechanisms noted included fire, iron, friction, chemical, electric, hair straightener, and radiator.

The average time to healing was 10 days (range 2-28 days; Table 1); 86.5% (n=45) of patients were conservatively managed. Conservative management varied depending upon the type of injury but included gentle cleansing of wounds without general anesthesia and twice-daily dressings with silver sulfadiazine, bacitracin, and/or petrolatum-based bandages and ointments; 97.8% of patients undergoing this method of management healed spontaneously, with one patient developing wound infection as a complication of this approach.

**Table 1:** Percentage of patient cohort versus days to healing

Days required for healing to occur	Percentage of the patient cohort
≤7	36.50%
8–14	46.20%
15–21	9.60%
>21	7.70%

**Figure 4:**
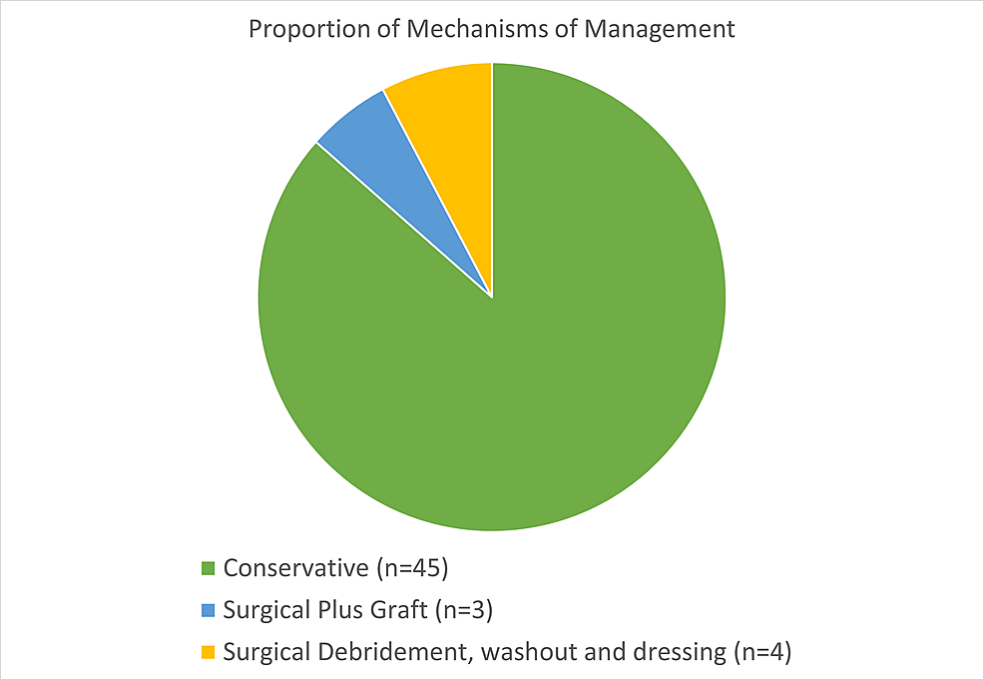
Pie graph representing the three management plans. Comparing conservative (dressings alone) with surgery and grafting and surgical debridement washout and dressing.

**Figure 5:**
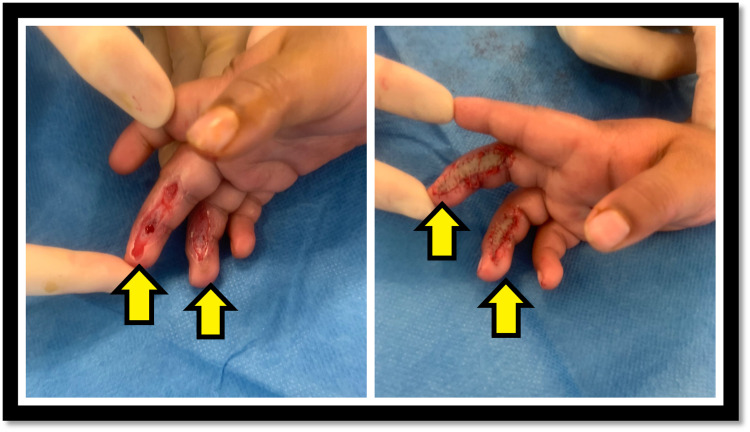
Pre (left) and intra (right) operative images of friction burn to the right-hand middle finger and ring finger sustained from accidental injury with a treadmill, managed with debridement and full-thickness skin grafts. Arrows point at the affected fingers on the right hand.

Surgical management was required for 13.5% (n=7) of patients (Figure 4). Indications for surgical intervention included the failure of an open wound to close within two to three weeks of conservative therapy, early contracture release, and significant exposure of flexor tendon. Of these seven patients, four had surgical debridement of burn tissue, washout, and dressing, and the remaining three had an excision and grafting with thick split-thickness skin grafts. Of these three patients, one patient had to undergo secondary reconstruction with a full-thickness skin graft. The infection rate was <3% among the whole series, and broad-spectrum antibiotic prophylaxis was given to patients requiring surgical intervention. Figure 5 shows an example of deep friction burns to the digits which required debridement and full-thickness skin grafting. Only one patient reported post-injury complication and underwent secondary reconstruction. Time to complete healing was independent of hospital stay.

## Discussion

Within the paediatric plastic surgery outpatient department, the most common part of the human body receiving injuries is the hands [7]. Our study, similar to other studies [8], documented that young children are more prone to injuries on their hands owing to their curious nature. Studies on hand burns are of the utmost importance for evaluating the prevailing preventive measures in society for burn injuries. This further helps in devising strategies for treatment and implementing preventive practices in every household, therefore, relieving tertiary care hospitals from the burden of burn injuries. Therefore, our retrospective study aimed to investigate the isolated burns to the hand, including the causes, demographic data, management, and outcome.

In this review, 52 paediatric patients affected with isolated hand burn injuries were taken for study and were followed for a period of up to three years. Most of the patients were managed conservatively whereas a few required surgical intervention.

Based on electronic clinic letters, wounds that required 21 days or longer to heal had scarring (hypertrophic), contractures, and intense tenderness as some of the later stage presentations. Three of these patients underwent surgical repair and reconstruction with split-thickness skin grafting, following the experience of seniors. According to Prohaska and Cook [9], currently, there is no convincing evidence to suggest that thick split skin grafting is inferior to full-thickness grafting and that these have comparable results once healed.

The initial assessment of a hand burn is incredibly vital as it can determine the course of management in the patient. Some of the significant aspects to consider in this assessment, as stated by Argirova and Hadzhiyski [10], include the mechanism of injury, the character of the burning agent, the contact time, previous hand diseases, and assessment of perfusion. All these factors greatly determine the initial management, future referrals, conservative management, and/ or surgical intervention as is also evident from our study.

According to Jozsa et al. [11], paediatric hand burn injuries more commonly occur in families with lower socioeconomic status, and with these patients, there is also a greater risk of subsequent infection. Although this aspect was not analysed in this study, going forwards, this could be an interesting demographic to look into in detail in further studies, using the Scottish Index of Multiple Deprivation tool as an indicator.

From our study, it can be confirmed that palmar surface paediatric burns are more common than dorsal surface burns. Whilst there is literature available looking at long-term sequelae in palmar surface burns, there is limited availability of these data for dorsal surface burns. This is an area that can be studied in further detail once a larger dataset is accumulated, likely as a multi-centre study.

Data in this study revealed contact with the hob and other thermal causes (including irons and radiators) to be the most common mechanisms for burns in this population. This was also found to be the case in studies by Brownlee et al. [12] and Krishnamoorthy et al. [13]. The kitchen was therefore a common site of injury and highlights the importance of increasing awareness and developing strategies to prevent injuries like these occurring at home.

To conclude, it has been found that the majority of patients in this study completely healed with primarily conservative measures of management and through regular check-ups. Isolated hand burns in the paediatric population present a low rate of sequelae and palms are the most common area of burn in this demographic. With further research into hand burns of the dorsum and fingers, the occurrence of late sequelae can be compared in the different groups to help determine which cohort has a better prognosis long-term.

## Conclusions

Paediatric patient presenting with burn injuries is common and rapid assessment of a child with burn injuries is crucial for the best possible outcome. Accidental contact burns involving hands are predominant in children involving mostly the left-hand palm, regardless of hand dominance. The treatment of burn injuries in children is multimodal and can range from simple dressing care to surgical interventions, involving surgical excision of the burn and reconstruction. Most paediatric hand burns healed completely with primarily conservative measures of dressing care and regular checkups within two weeks. However, prompt stabilization, resuscitation, and transfer to a definitive care facility are paramount. We also observed very low sequelae of isolated hand burns in paediatric population. Finally, contact burns in hand among our target population can be prevented by taking necessary safety measures, as accidental contact with hob was the most common etiology of these injuries in children.
